# Effect of virtual reality self-counseling with the intimate other avatar

**DOI:** 10.1038/s41598-024-65661-6

**Published:** 2024-07-04

**Authors:** Yuko Yamashita, Tetsuya Yamamoto

**Affiliations:** 1 Graduate School of Sciences and Technology for Innovation, Tokushima University, Tokushima, Japan; 2 Research Fellow of Japan Society for the Promotion of Science, Tokyo, Japan; 3 Graduate School of Technology, Industrial and Social Sciences, Tokushima University, Tokushima, Japan

**Keywords:** Disease prevention, Quality of life, Human behaviour, Computer science

## Abstract

Virtual reality self-counseling (VR-SC) is considered an effective approach for addressing mental health problems. Previous studies have shown the effectiveness of VR-SC using Sigmund Freud’s avatar as the counselor. However, considering that virtual reality (VR) enables embodied perspective-taking of another person, VR-SC using the avatar of a person who cares about the participant (an intimate person), such as a family member or friend, is considered effective because it could create warm attitudes toward the participants themselves. In this study, 60 undergraduate and graduate students were split into three conditions: VR-SC with intimate persons, VR-SC with Freud, and a control group. The intervention effects were then compared. The results showed that VR-SC with an intimate person was the most effective in improving anxiety symptoms. These results may be attributed to accepting and affirming oneself from the perspective of the intimate person’s avatar and counseling oneself. This study is significant in that it is the first to conduct VR-SC with the avatar of an intimate person and compare the effects with Freud’s avatar. More importantly, it showed that the same VR-SC method could have different effects depending on the avatar of the counseling partner.

## Introduction

Mental health problems have become increasingly prevalent in the modern world. According to the World Mental Health Japan Survey 2nd (2013–2015), the lifetime prevalence of any common mental disorder is 22%^1^. Mental health problems are associated with various issues, such as poor achievement in school^2^, low labor productivity^3^, and suicidal behavior^4,5^, causing significant psychological distress. Therefore, effective approaches are needed to address mental health problems.

Various types of psychological therapies provided by professionals and social support from people such as family members and friends have been proven to be effective^6–10^. However, many people hesitate to use conventional mental health services or seek support from those around them because of concerns about social stigma, financial burden, disclosure of confidential information, and inconvenience to those with whom they talk^11–19^. Thus, many people have psychological problems but are unable to obtain sufficient support from professionals and others around them, and as a result, the problem often worsens.

Therefore, in this study, we focused on the potential application of virtual reality (VR) to help participants deal with their psychological difficulties. Virtual reality involves a multisensory integration and viewing the virtual body from the first-person perspective, thereby creating the body ownership illusion, which makes one experience the avatar’s body as if it were one’s own. Previous studies have shown that the different avatars experienced in VR have different effects on people^20^. For example, one study indicated that embodiment of light-skinned participants in a dark-skinned avatar through the use of VR significantly reduced implicit racial bias against dark-skinned people^21^. Another study involved an experiment where participants played a West-African Djembe hand drum while immersed VR. Participants were represented either by a casually dressed dark-skinned virtual body, or by a formal suited light-skinned body, and the findings revealed that those with the former representation showed significant increases in their drumming movement patterns than the latter^22^. Furthermore, the embodiment of a child’s avatar induces the overestimation of the sizes of objects^23^, and the embodiment of an avatar of Albert Einstein improves performance on a cognitive task and decreases implicit bias against older people^24^. These studies have shown that the avatar experienced in VR has a significant impact on our cognition and behavior.

Previous studies have attempted to examine the effectiveness of VR self-counseling (VR-SC) by utilizing these features of VR. In Osimo et al.^25^, participants alternately switched between the body of their own avatar, who described a personal problem, and the body of the counselor’s avatar, who offered them counseling. That study presented two conditions—In one condition, Sigmund Freud, the founder of psychoanalysis, was used as the avatar of the counselor, and in the other condition, the counselor appeared in the avatar of the participant themselves. The participants reported better moods and greater happiness after interacting with Freud than after interacting with themselves. These results revealed that different avatars of counselors have different effects. In addition, it was also shown that the stronger the body ownership illusion, the greater the improvement in mood. Furthermore, other studies have reported that the process of alternating between the bodies of two avatars in VR-SC contributes to the effective resolution of problems^26^. Thus, previous studies have shown that the body ownership illusion and perspective-taking of another person in VR-SC play important roles in helping individuals navigate and deal with their psychological problems.

Virtual reality self-counseling is considered an effective approach because it provides accessibility to individuals who are reluctant to seek support from others. Participants can experience the treatment alone without the presence of another person, such as a therapist. However, the effect of VR-SC when a person other than Freud is presented as the counselor’s avatar remains unclear^25^. Considering that VR can afford embodied perspective-taking of another person, it is assumed that having a person who cares about the individual, such as a family member, close friend, or lover (hereafter, referred to as “intimate person”), as a counseling partner may also be effective. Consistent with this idea, in cognitive behavioral therapy, it is recommended that patients use the perspectives of family, close friends, and others when examining maladaptive automatic thoughts^27^. Thus, it is thought that taking the perspective of an intimate person in VR would enable individuals to view their problems objectively. Furthermore, it is assumed that speaking to an intimate person in VR-SC would create a sense of comfort and acceptance for the participant, which may facilitate the improvement of psychological problems after the experience. Hence, a VR-SC with an intimate person as the counselor’s avatar is expected to improve the participant’s mental health.

Therefore, to examine the effects of VR-SC with intimate persons on the improvement of mental health, this study compared the intervention effects of VR-SC with an intimate person and VR-SC with Freud. In addition, as a control group was not established in the previous studies^25,26^ that examined the effects of VR-SC, this study included a control group in addition to the above two groups to control for confounding factors, such as improvement and recovery facilitated by the passage of time.

**Figure 1:**
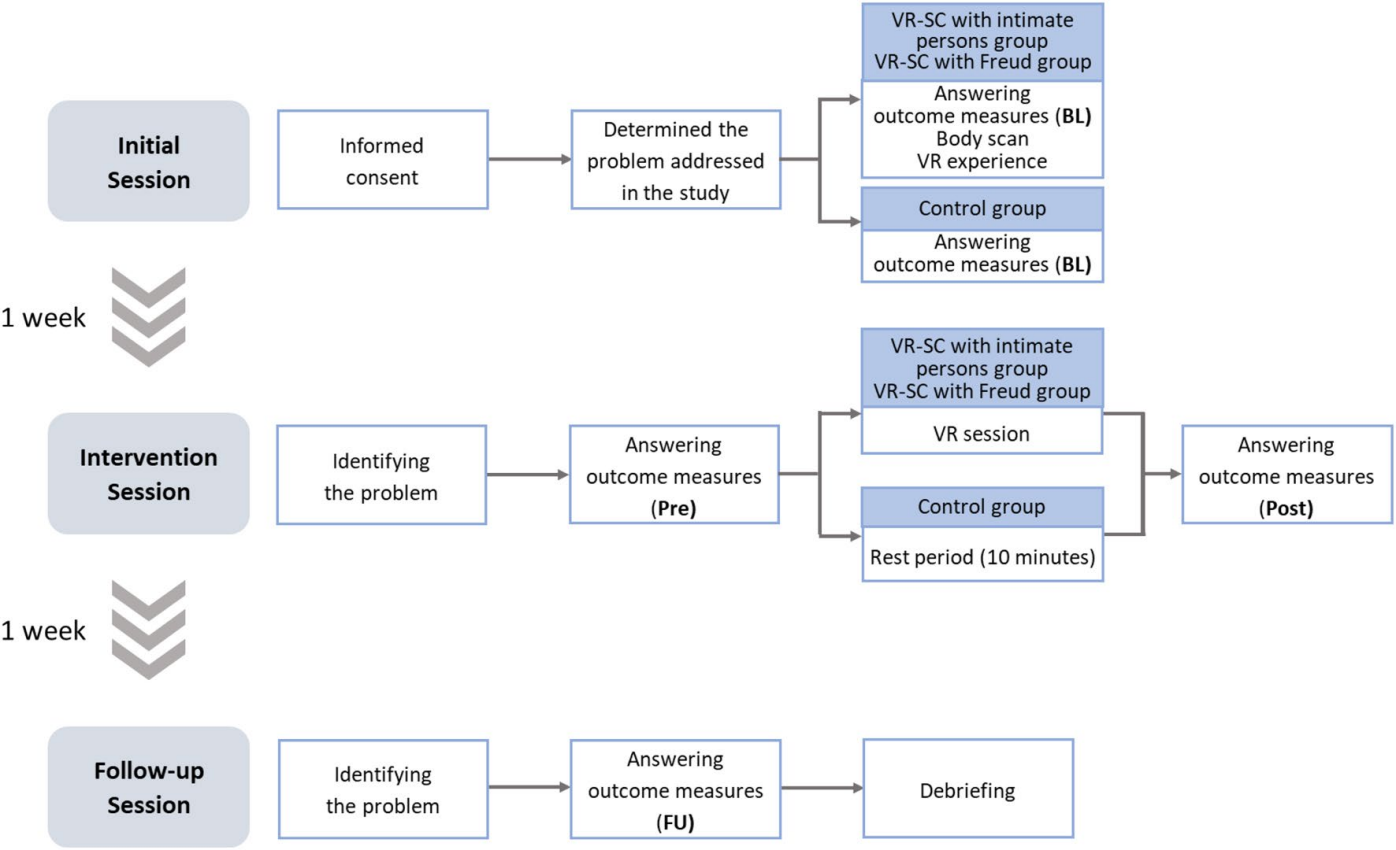
The flow of the experiment. *BL* baseline time point, *Pre* pre-intervention time point, *Post* post-intervention time point, *FU* follow-up time point.

Sixty undergraduate and graduate students (23 men, 37 women; mean age = 21.00 years; *SD* = 1.90) participated in this study. They were divided into three groups of 20 people each: VR-SC with intimate persons group (“IP group”), VR-SC with Freud group (“Freud group”), and a control group that underwent a 10 min rest period instead of an intervention. The study compared the intervention effects on the three groups. As an outcome measure, participants rated the discomfort level of their problem addressed in this study on an 11-point scale ranging from 0 (peace, serenity) to 10 (insupportable). The Patient Health Questionnaire-9 (PHQ-9^28,29^) and the generalized anxiety disorder-7 (GAD-7^30,31^) were also used to measure the degree of depressive and anxiety symptoms, respectively. As outcome measures for the problems, we used some of the measures used by Slater et al.^26^ (details are shown in Table 1; questions were modified to fit the measurement time point and intervention method). After the intervention or the rest period, the participants were asked about the changes in their problems caused by the interventions/rest period and their impressions of those experiences. To examine the validity of the VR-SC environment, the sense of presence and the body ownership illusion were measured (details are shown in Supplementary Tables S2 and S3; scales from previous studies^25,26^ were modified and used to fit the procedures in this study). In addition, the degree of perspective-taking of the counseling partner was rated on an 11-point scale from 0 (not at all possible) to 10 (very possible) for participants in the VR-SC groups. Participants visited the laboratory three times: for the initial session, intervention session, and follow-up session (Fig. 1). The outcome measures were measured at four time points: baseline (BL) in the initial session, pre-intervention (Pre) and post-intervention (Post) in the intervention session, and follow-up (FU) in the follow-up session (details of the measurement points for each scale are described in the "Methods" section). These procedures were followed to compare the effects of the interventions.

**Table 1:** Outcome measures related to the problem.

Variable	Questionnaire item	Scale
*Help*	(Pre) How much did the previous participation (initial session) in the study help you as regards to the problem? (Post) How much did the counseling/rest period help you as regards to the problem? (FU) How much did the counseling/rest period help you as regards to the problem?	0 = not sure 1 = made the problem a lot worse 2 = made the problem somewhat worse 3 = made no difference 4 = made the problem somewhat better 5 = made the problem a lot better
*Understand*	(Post) I think that, after this experience in the counseling/rest period, I am able to better understand my problem (FU) I think that after the previous experience in the counseling/rest period, I am able to better understand my problem	− 3 = least agreement − 2 − 1 0 1 2 3 = most agreement
*NewIdeas*	(Post・FU) I think I could have new ideas on how to solve my problem	
*BetterControl*	(Post・FU) I feel that I control my problem better	
*Helped*	(Post) This dialogue/rest period helped me to have a new perspective on my problem (FU) The previous dialogue/rest period helped me to have a new perspective on my problem	

*Pre* pre-intervention time point, *Post* post-intervention time point, *FU* follow-up time point.

## Results

### Demographic characteristics and descriptive statistics for each outcome measures

Table 2 presents the participants’ demographic characteristics, and Table 3 shows the descriptive statistics for each variable at the four time points. The persons identified by the IP group as those who cared about them (intimate others) and offered them counsel were: 11 friends (best friends), 6 lovers, 2 family members (including 1 mother and 1 sister), and 1 academic senior. The psychological problems addressed in this study are presented in Supplementary Table S1.

**Table 2:** Demographic characteristics and VR experience of the participants.

	VR-SC with intimate persons group (*n* = 20)		VR-SC with Freud group (*n* = 20)		Control group (*n* = 20)	
	Men	Women	Men	Women	Men	Women
Sex	7	13	8	12	8	12

	M	SD	M	SD	M	SD
Age (years)	21.00	1.34	21.45	2.58	20.55	1.50
Number of prior VR experiences (times)	0.90	1.59	1.10	2.69	–	–
Prior VR experience time (minutes)	27.75	51.97	70.65	273.37	–	–

**Table 3:** Descriptive statistics for the outcome measures.

	Group	BL		Pre		Post		FU	
		M	SD	M	SD	M	SD	M	SD
Degree of perspective-taking of the counseling partner	VR-SC with intimate persons group	–	–	–	–	6.80	1.99	–	–
	VR-SC with Freud group	–	–	–	–	5.65	2.39	**-**	-
Discomfort level of the problem	VR-SC with intimate persons group	5.75	1.55	5.50	1.50	3.70	2.11	4.10	1.83
	VR-SC with Freud group	5.55	1.61	5.00	1.59	4.05	1.76	3.90	1.71
	Control group	5.50	2.09	4.95	1.76	4.50	1.91	5.40	1.64
PHQ-9	VR-SC with intimate persons group	4.05	3.03	3.10	2.45	–	–	2.45	2.21
	VR-SC with Freud group	5.60	3.50	4.05	3.20	–	–	4.35	3.59
	Control group	6.70	3.99	6.20	4.36	–	–	5.20	4.09
GAD-7	VR-SC with intimate persons group	4.05	3.05	2.45	2.06	–	–	1.30	1.26
	VR-SC with Freud group	3.65	3.45	3.50	3.19	–	–	3.45	3.38
	Control group	4.35	3.10	3.75	3.70	–	–	3.55	3.15
Outcome measures related to the problem									
*Help*	VR-SC with intimate persons group	–	–	2.85	0.67	4.25	0.44	4.15	0.37
	VR-SC with Freud group	–	–	3.00	0.79	3.70	1.30	3.95	1.05
	Control group	–	–	3.10	0.91	2.85	1.31	3.15	0.88
*Understand*	VR-SC with intimate persons group	–	–	–	–	1.85	0.81	1.90	0.85
	VR-SC with Freud group	–	–	–	–	2.00	0.86	1.90	0.72
	Control group	–	–	–	–	− 0.30	1.42	0.00	1.59
*NewIdeas*	VR-SC with intimate persons group	–	–	–	–	1.45	1.23	1.40	1.10
	VR-SC with Freud group	–	–	–	–	1.15	1.53	1.25	1.29
	Control group	–	–	–	–	− 0.40	1.73	− 0.40	1.60
*BetterControl*	VR-SC with intimate persons group	–	–	–	–	1.05	0.69	1.35	0.88
	VR-SC with Freud group	–	–	–	–	1.40	1.14	1.50	0.76
	Control group	–	–	–	–	− 0.25	1.55	0.20	1.24
*Helped*	VR-SC with intimate persons group	–	–	–	–	1.90	0.97	2.10	0.91
	VR-SC with Freud group	–	–	–	–	1.70	1.17	2.05	0.89
	Control group	–	–	–	–	− 0.35	1.66	− 0.25	1.71

**Figure 2:**
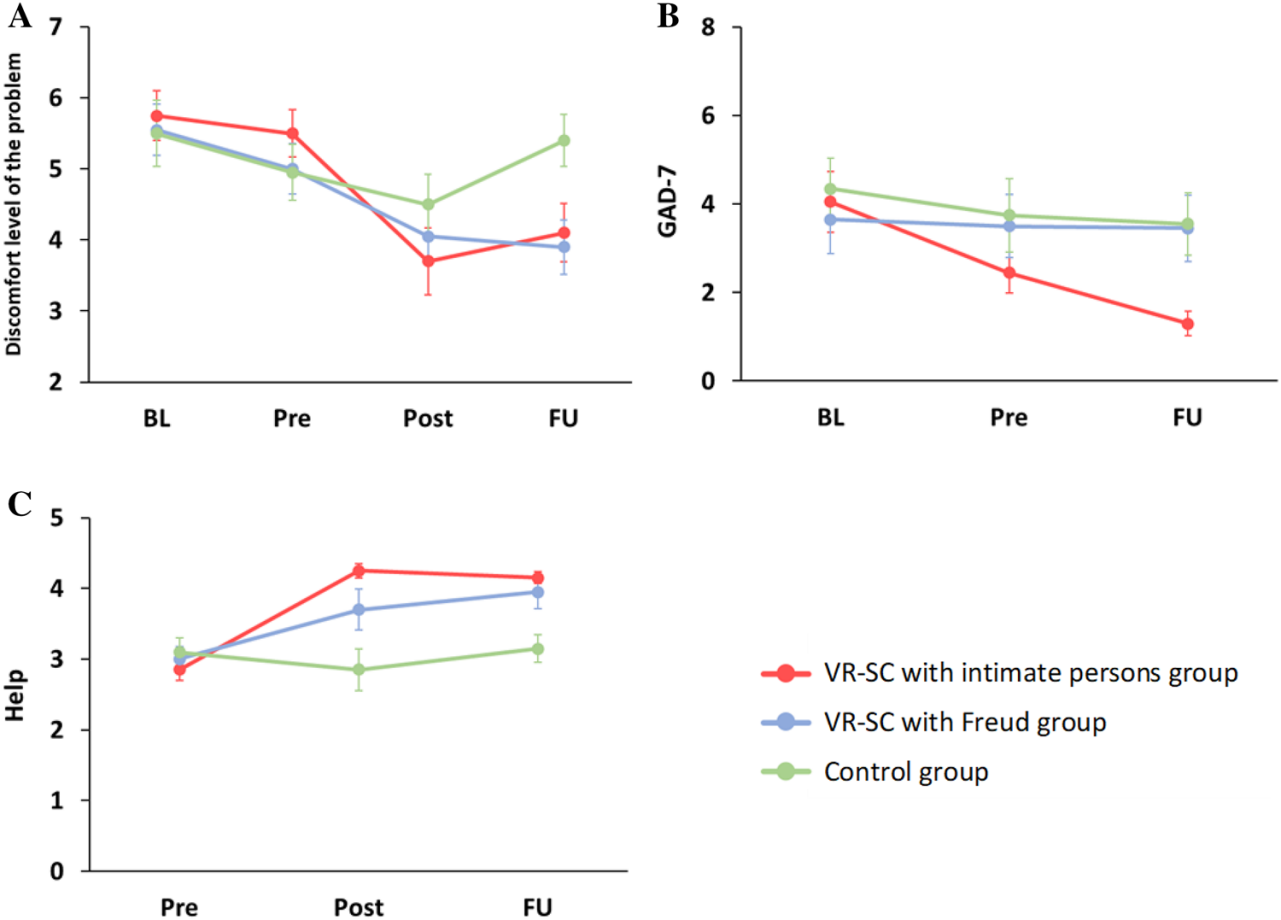
Change in outcome measures. (**A**) Discomfort level of the problem. (**B**) GAD-7 (generalized anxiety disorder-7). (**C**) The *Help* variable (the extent of improvement in the participant’s problem due to the intervention or rest period). Error bars represent the standard errors.

The graphs of these measures are shown in Fig. 2 and Supplementary Figs. S1–6.

*BL* baseline time point, *Pre* pre-intervention time point, *Post* post-intervention time point, *FU* follow-up time point, *PHQ*-*9* patient health questionnaire-9, *GAD*-*7* generalized anxiety disorder-7.

### Validity of the VR environment

The mean and standard deviation for each question in the scales for the sense of presence, social presence, body ownership illusion, and sense of agency are shown in Supplementary Tables S2 and S3. The values confirmed that the VR experience was successful in creating the sense of presence, social presence, body ownership illusion, and sense of agency.

To examine the difference between the means of the IP and Freud groups regarding the degree of perspective-taking of the counseling partner, we conducted an unpaired t-test. While no significant differences were found between the two groups, the higher effect size indicated that the IP group had a higher degree of perspective-taking than the Freud group (*t* (38) = 1.65, *p* = 0.106, *d* = 0.52).

### Outcome measures

#### Discomfort level of the problem

Regarding the discomfort level of the problem, the interaction between group and time was significant (*F* (5.17, 147.28) = 4.39, *p* < 0.001, *ηp*^*2*^ = 0.133; Fig. 2A). The simple main effect of group at FU was significant (*F* (2, 57) = 4.44, *p* = 0.016), and the discomfort level of the Freud group at FU was lower than that of the control group (*p* = 0.024). In addition, the simple main effects of time for all groups were significant (IP group, *F* (2.58, 147.28) = 19.12, *p* < 0.001; Freud group, *F* (2.58, 147.28) = 11.47, *p* < 0.001; control group, *F* (2.58, 147.28) = 3.91, *p* = 0.010). First, in the IP group, the discomfort level was significantly reduced at Post and FU compared to BL and Pre time points (*ps* < 0.001). In the Freud group, the discomfort level was significantly reduced at Post and FU compared to BL and Pre time points (*ps* ≤ 0.008). In the control group, the discomfort level increased significantly at FU compared to that at the Post time point (*p* = 0.044).

#### Depressive and anxiety symptoms

For PHQ-9 scores, there was no significant interaction between group and time (*F* (4, 114) = 0.77, *p* = 0.547, *ηp*^*2*^ = 0.026). However, for GAD-7 scores, the interaction was significant (*F* (4, 114) = 3.55, *p* = 0.009, *ηp*^*2*^ = 0.111; Fig. 2B), and the simple main effect of group at FU was significant (*F* (2, 57) = 4.22, *p* = 0.019). The GAD-7 scores of the IP group at FU were lower than those of the control group (*p* = 0.038). Moreover, the simple main effect of time on the IP group was significant (*F* (2, 114) = 15.12, *p* < 0.001). The scores were significantly reduced at Pre compared to BL (*p* = 0.010), and at FU compared to BL and Pre in the IP group (*p* < 0.001; *p* = 0.043).

#### Outcome measures related to the problem

As outcome measures for the problems, we used the scale in Table 1. Of these variables, only the results for the *Help* variable, for which significant interactions were found, are described here; the details of the results for the other variables are presented in the “Supplementary Results”. The *Help* variable showed a significant interaction between group and time (*F* (3.07, 87.46) = 7.25, *p* < 0.001, *ηp*^*2*^ = 0.203; Fig. 2C) and significant simple main effects of group at the Post and FU time points (*Fs* (2, 57) ≧ 8.28, *ps* < 0.001). At the Post time point, the scores of the IP group were significantly higher than those of the control group (*p* < 0.001). At the FU time point, the scores of the IP and Freud groups were significantly higher than those of the control group (*p* < 0.001; *p* = 0.009). In addition, there was a significant simple main effect of time in the IP and Freud groups (*Fs* (1.53, 87.46) ≥ 9.18, *ps* < 0.001). In the IP group, scores increased significantly at Post and FU compared to the Pre time point (*ps* < 0.001). The VR-SC with Freud group also showed a significant increase in scores at Post and FU time points compared to the Pre time point (*p* = 0.048;* p* < 0.001). Only in the IP group, all 20 participants reported that their problems improved at both Post and FU time points. On the other hand, in the VR-SC with Freud group, 18 participants reported that their problems improved at both the Post and FU time points. In the control group, six participants reported that their problems improved at both the Post and FU time points.

Supplementary Tables S4 and S5 show the number of participants who reported that the intervention or rest period caused a change in their problems at the Post and FU time points.

## Discussion

The purpose of our study was to examine the intervention effects of VR-SC with intimate persons based on the data obtained from the VR-SC with intimate persons, VR-SC with Freud, and control groups.

First, the descriptive statistics of the responses to the measures of presence, social presence, body ownership illusion, and sense of agency confirmed that these sensations were experienced, ensuring the validity of the VR-SC environment.

The results of our study indicated that VR-SC with both an intimate person and Freud was effective in improving the discomfort level of problems.

Only the IP group had significantly lower GAD-7 scores after the intervention, and significantly lower scores than the control group at the FU time point. Therefore, in terms of the improvement of anxiety symptoms, intimate persons were found to be more effective than Freud as counseling partners. Regarding the reasons for the change in their problems, the IP group provided reasons such as “I thought my counseling partner would tell me I was doing my best without denying me” and “By assuming the perspective of the counseling partner, I was able to have a dialogue that reassured me”. Therefore, it is thought that the participants’ anxiety levels were reduced by the experience of receiving warm acceptance and affirmation from the perspective of the avatar of the intimate person who cared about them.

Regarding the problems addressed in the study, the results showed that following the intervention, both IP and Freud groups, compared to the control group, had a deeper understanding and increased sense of control, acquired a new perspective, and discovered solutions for their problems. Interestingly, in the IP group only, all participants reported an improvement their problems at Post and FU time points. This suggests that even with the same VR-SC method, the effect on the improvement of the problem differs depending on the avatar of the counseling partner.

There are two possible reasons for these results. First, unlike counseling with Freud, in counseling with the avatar of the intimate person, it is easier to imagine how they will respond because they are well-known to participants, thus facilitating perspective-taking in VR. In fact, our results indicated that the IP group had a higher degree of perspective-taking than the Freud group. In addition, participants in the Freud group reported that it was difficult for them to listen and speak as Freud, suggesting that some of them experienced difficulty acquiring Freud’s perspective. Therefore, the IP group may have been able to more easily obtain the perspective of the counselor and more smoothly engage in self-dialogue, which may have helped them view their situation objectively from the perspective of the other person. These effects might be related to the fact that all participants in the IP group showed improvement in their problems. The second reason is the enjoyment of the intervention method. After the intervention, both the VR-SC with intimate persons and Freud group reported that they enjoyed the VR experience. Especially in the IP group, many participants smiled when they saw the avatar of the intimate person during the VR session. One of the participants from the IP group said: “Since the avatar resembled both me and my lover, I enjoyed the same atmosphere in the VR space as when we actually met.” Thus, it appears that having an intimate person as a counseling partner made the VR-SC experience more enjoyable and pleasant for the participants, which may have elevated the quality of self-therapy and helped improve their problems. Thus, VR-SC with intimate persons could be considered an effective method based on its ability to put the participant at ease and generate a more positive and receptive mood as the participant attempts to speak about a painful situation.

Our results suggest that VR-SC with an intimate person is an effective approach for maintaining and improving mental health. Participants who experienced the VR-SC commented, “I am not good at talking about my problems to anyone, but it was easy to talk about them because the other person was a person in the virtual space,” and “I thought it was a good way for people who hesitate to talk their problems with others.” Thus, VR-SC is an effective approach for reducing psychological problems, especially for those who are reluctant to seek support from people around them. Furthermore, some of the participants in the IP group reported changes in their feelings after the intervention: “I was worried and wondered if I should not talk about it, but now I feel like I should talk to a counseling partner” and “I realized that I do not have to worry alone.” Thus, the results indicate that VR-SC with an intimate person may facilitate help-seeking behaviors in the real world.

Virtual reality self-counseling is a counseling method that can be used any time one needs counseling or even when one is unable to consult the intended person in the real world due to physical distance or time issues. Furthermore, it has recently been reported that psychological stress has been increasing due to the spread of COVID-19^32^, and it is recommended that people refrain from interpersonal contact. Under such circumstances where interpersonal contact is limited and it is difficult to counsel with others, the use of VR-SC may help alleviate psychological stress.

Our study was the first to conduct VR-SC with an intimate person and compare the effects with those of VR-SC with Freud. As a future prospect, the effect of VR-SC with various other avatars of counseling partners is expected. It is believed that the people with whom one wishes to talk about problems differ depending on the nature of the problems and personal relationships. For example, if a mother is related to the participant’s problem, VR-SC with the mother’s avatar could reinforce a participant’s negative emotions and the intervention would not function effectively. As this case shows, VR-SC with intimate persons is not necessarily effective in all cases. Therefore, if technology is realized in the future that allows people to select the person they want to consult with and conduct VR-SC with that person whenever they want, it is possible that intervention effects beyond those obtained in the present study will be demonstrated.

Finally, this study has several limitations. First is the issue of the selection method of the participants. The study population consisted of those who had a distress level of 2 or higher for their current problems. However, as relatively healthy undergraduate and graduate students participated in this study, the change in scores on the outcomes due to the intervention was small, suggesting that there was no improvement in depressive symptoms. Different results may be obtained by including participants with higher discomfort levels and depressive tendencies in the study. In addition, our study prioritized securing the number of participants; those who could obtain the cooperation of their intimate person were mainly classified into the IP group. Hence, complete random allocation was not possible. As such, future studies should examine the effect of the intervention using a complete random assignment design. The second limitation is the number of interventions. More than half of the participants who experienced VR-SC had never experienced VR before, and all were new to VR-SC. Participants also commented that they could not get used to VR-SC initially. Therefore, further intervention effects may be obtained by establishing a counseling method among participants through multiple interventions. The third is the issue of the counseling partner’s voice in the VR-SC. In this study, statements made by the participants from their own avatars and from the avatars of their counseling partners were set to be played back in the participants’ own voices. However, several participants reported that they felt strange hearing their own voices coming from their counselor’s avatar, which might have reduced their sense of immersion. Therefore, it would be desirable to appropriately process the voice coming from the counselor’s avatar. Another possibility is using counselors of the same gender as the participant. The effects of such modifications could be investigated in future studies.

Our results demonstrated the effectiveness of VR-SC and suggested that it may be the paradigm that can actually be utilized to improve psychological difficulties. However, the number of studies and evidence showing the effectiveness of VR-SC is not sufficient at this time. Therefore, the target population for whom VR-SC can be effectively applied is also unclear. For example, one study has reported that patients with anorexia nervosa experienced greater anxiety about their body and have lower levels of full-body illusion than healthy controls, when they owned a virtual body that had their silhouette and body mass index^33^. Patients with anorexia nervosa may face difficulties with the perspective-taking aspect of avatars, and discussing their own problems in VR-SC comfortably. Thus, VR-SC could not function effectively for some subjects, such as in cases of patients with mental disorders, and therefore requires caution in its adaptation. Considering the above, at this point, VR-SC should be used for healthy people who do not suffer from mental disorders, but face problems in their daily lives, such as the subjects in our study. It might be appropriate to position and develop VR-SC as the method to prevent the onset of serious mental health problems and mental disorders. Including such limitations and ways to appropriate utilization of the intervention, we must wait for more research findings to be accumulated in the future.

## Methods

### Participants

Participants were recruited by visiting university classes, posting recruitment posters on university bulletin boards, spreading the posters through social networks, and using snowball sampling. Of the 138 applicants, those who currently had personal problems and felt that their problems would persist for some time in the future, had a discomfort level of 2 or higher (on a scale of 0 to 10), and were not currently using counseling or any other psychological treatment were eligible for the study. In addition, because of safety considerations when conducting the VR experiment, we excluded participants with the following conditions: (1) pregnancy, (2) epileptic seizures, (3) visual impairment, (4) mental disorders, (5) weak heart, and (6) other serious medical conditions. Applicants who met the criteria were individually contacted and asked to participate in the study. Among these applicants, those who agreed to participate were asked to visit the laboratory. Participants who were able to obtain the cooperation of intimate persons were mostly classified into the IP group, while the remaining participants were randomly classified into either the Freud or control group. In the classification, stratified block randomization was used to ensure that the discomfort levels of the problems in each group were similar.

Two participants who met the diagnostic criteria for major depressive episodes as per the Japanese version of the Mini-International Neuropsychiatric Interview (MINI^34,35^) were asked to discontinue participation in the experiment to ensure their safety. In addition, one international student was excluded from the study and one participant dropped out.

Finally, 60 undergraduate and graduate students (23 men, 37 women; mean age = 21.00 years; *SD* = 1.90) who participated in the experiment until the end were included in the analysis. Twenty participants were classified into each of the three groups: VR-SC with intimate persons, VR-SC with Freud, and control groups.

To conduct VR-SC with an intimate person, we asked the intimate persons of the participants to cooperate in the body scan to create a 3D avatar. Those intimate persons who indicated their willingness to cooperate were asked to come to the laboratory before the day of the intervention session to create their avatars. At the time of their visit, they were informed about the study, and those who agreed to cooperate underwent a body scan and were given an honorarium.

This study was approved by the Research Ethics Committee of the Graduate School of Social and Industrial Science and Technology, Tokushima University (approval number 228), and all experiments were performed in accordance with the committee’s guidelines and regulations. Informed consent was obtained from each individual for their participation in this study and for the publication of their photograph and avatar in the figures of this paper.

### Materials

**Figure 3:**
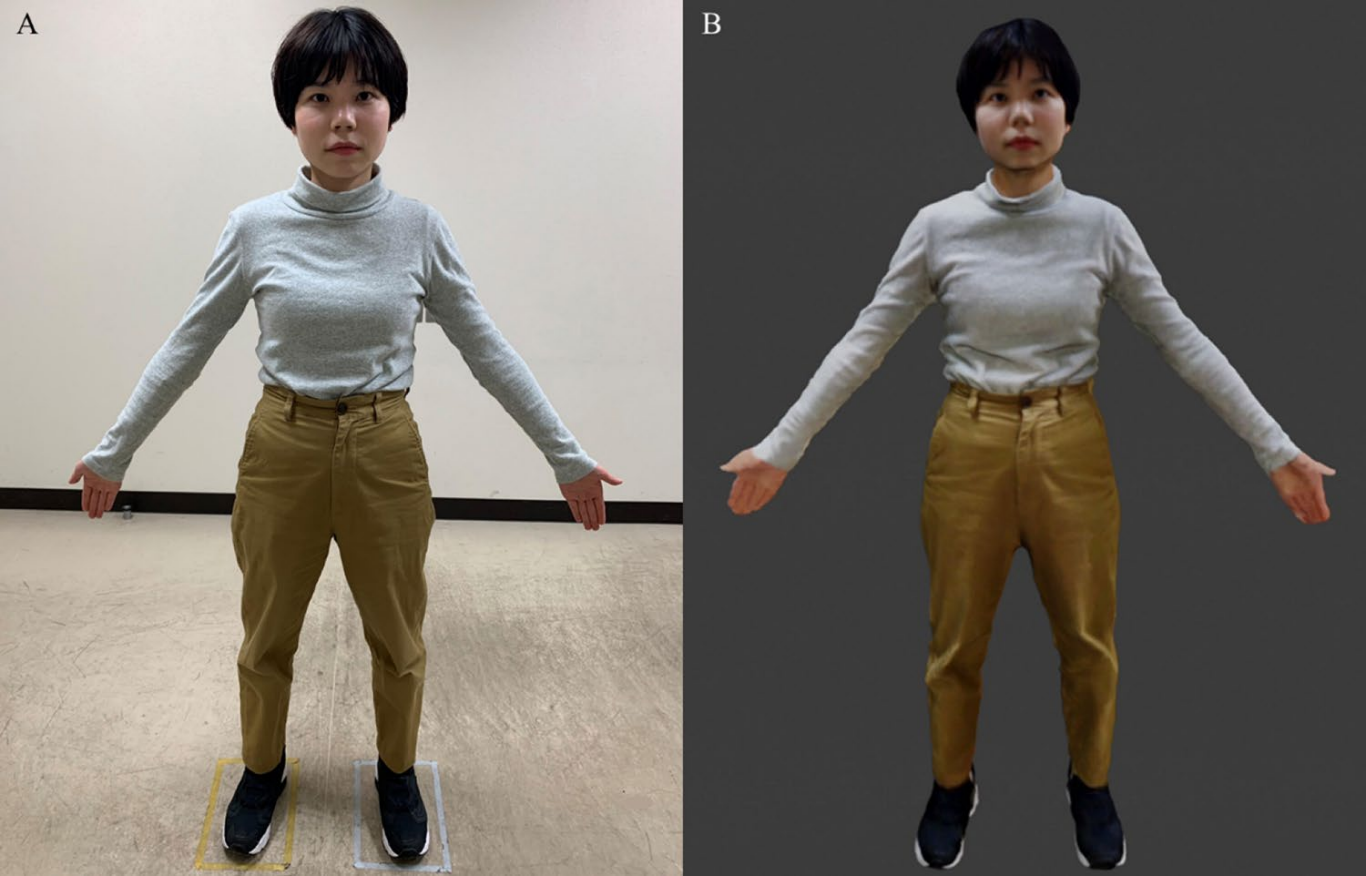
The 3D avatar created in this study. (**A**) A photo of the actual participant. (**B**) A 3D avatar of the participant created through body scanning, among other methods.

To conduct VR-SC, we created 3D avatars of the participants. For body scanning to create 3D avatars, we used an iPad 6th generation, Structure Sensor Mark II, which is a 3D scanner, and itSeez3D, which is an application for 3D scanning. To process the 3D data after scanning, we used Blender, an integrated 3DCG software, and Mixamo, which is a web service for 3D model animation production. Figure 3 shows the 3D avatar created in this study.

The VR space was created using the Unity 2019.3.12f1 game engine. We used an Oculus Rift CV1 head-mounted display (HMD) and Oculus Touch controllers. The HMD and two-handed controller were tracked using an Oculus Sensor. The OMEN HP 17-cb0004TX, a gaming laptop PC, was used to run the computer programs. The details of the equipment used in this study and the processes are described in the “Supplementary Methods” section.

### Procedure

Participants visited the laboratory three times: for the initial session, intervention session, and follow-up session. Outcomes were measured at four time points: BL in the initial session, Pre and Post in the intervention session, and FU in the follow-up session. Figure 1 illustrates the flow of the three sessions.

#### Initial session

Participants arrived at the laboratory and were briefed about the experiment before they signed the consent form. During the briefing, participants in the Freud group were provided with information about Sigmund Freud to familiarize them with him. We informed them that he was a famous psychologist and the founder of psychoanalysis and explained with emphasis that he was an expert in mental problems. Participants were confirmed to have no current major depressive episodes using the MINI, and then selected the psychological problems they wanted to address in this study. At this time, participants were identified as currently distressed (discomfort level of 2 or higher), and the distress was likely to persist for at least the next two weeks. They were then asked to answer questions about their problems (BL measurement). After answering the questions, the VR-SC groups underwent body scans to create 3D avatars. While the scanned data were being processed, the participants were asked to answer the remaining questions (BL measurement). Next, the participants of the VR-SC groups entered two VR spaces, a guidance session and a VR-SC session without avatars, for two minutes each to enable them to familiarize themselves with the VR space (details of each session are described below). During the VR experience, participants were instructed to spend their time freely looking and walking around the room. Participants in the control group did not go through the body scan and VR experience and were asked to answer the BL measurement questions together instead of in two separate sessions.

#### Intervention session

One week after the initial session, participants returned to the laboratory and were briefed on the procedure of the second session. At this time, the Freud group participants were again given brief information about Freud to reinforce their perception that Freud was an expert on mental problems. After the explanation, the problem addressed in the study was identified and participants were asked to respond to the questions (Pre measurement). The IP and Freud groups then underwent the VR session (detailed below), while the control group was given a rest period of 10 min. At the end of the VR-SC or rest period, participants were again asked to answer the questionnaire (Post measurement).

#### Follow-up session

One week after the intervention session, participants returned to the laboratory and were briefed on the procedure of the third session. The problem addressed in the study was then identified, and participants were asked to respond to the questionnaire (FU measurement). Finally, they were debriefed and rewarded.

### VR session

Participants in the VR-SC group underwent a guidance session, a VR-SC practice session, and a VR-SC session (See “Supplementary Movie”).

The guidance session was conducted to enhance the body ownership illusion and sense of agency in the avatars of the participants and their counseling partners (Supplementary Fig. S7). The participants entered an avatar of themselves standing in front of a mirror in the VR space, looked down at their own bodies, and moved their hands and necks according to the instructions heard through the headphone. Participants confirmed that the avatar’s body was located at the place where they were looking down and that their actual movements were linked to the avatar’s movements while looking in the mirror, thereby enhancing their sense of the avatar’s body as if it were their own body. Then, the same procedure was performed on the avatar of the counseling partner (intimate person or Freud) to increase the body ownership illusion and sense of agency in the avatar of the counseling partner. Each session lasted for approximately 5 minutes.

The purpose of the VR-SC practice session was to familiarize the participants with the VR space in which VR-SC was performed and the method of interaction (how to operate the controller). The participants conducted this session while seated on a chair. They first entered their own avatars and their counseling partners looked around the VR space, looked down at the avatars’ bodies, and moved their hands and necks while looking at the mirror located on the left side. After the participants became accustomed to the VR-SC space and the sensation of the seated avatar, they practiced the dialogue in the VR-SC. Participants first spoke freely from their own avatar, and when they finished speaking, they moved to the avatar of their counseling partner. They then heard the statements that they had just spoken in their own avatar through their headphones. After listening, they responded to the statements from the perspective of their counseling partner. After responding, they again moved to their own avatar and heard their own voice coming through their headphones, as they had just spoken in their counseling partners’ avatar. After listening, they responded to the statements from their own perspectives. In this way, they repeated the procedure of “speak, switch the avatar, listen to what the previous avatar said, speak…” until the participants became accustomed to conversation in VR.

**Figure 4:**
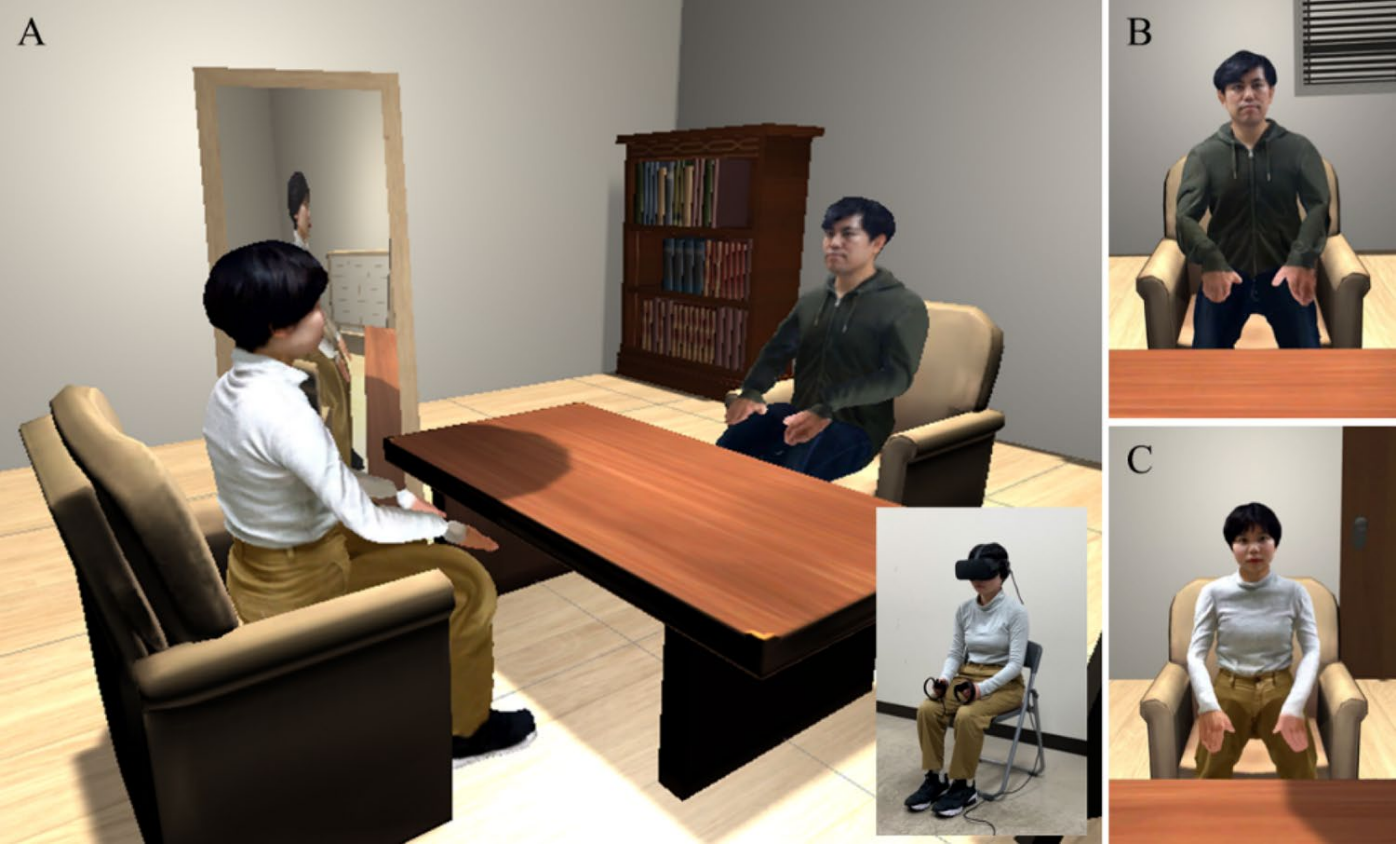
VR-SC with intimate persons. (**A**) Overhead view of the VR-SC and a participant. (**B**) The viewpoint of the participant after entering their own avatar. (**C**) The viewpoint of the participant after entering the avatar of the intimate person.

Then, in the VR-SC session, participants consulted about their problems using the same conversation procedure as in the practice session (Fig. 4, Supplementary Fig. S8). Before the VR-SC, we emphasized that when they entered the avatar of the counseling partner (intimate person or Freud), they should respond while being aware of the counseling partner’s perspective. In addition, we told them that they were allowed to speak freely, there was no right or wrong answer, there was no time limit (although we informed them that the maximum consultation time was one hour), and they could talk as much as they liked. When we explained the maximum counseling time to the participants, we told them to concentrate on the counseling without worrying about the time because the experimenter would report when the time was up.

After the VR experience, we presented questions from the simulator sickness questionnaire^36^ to confirm whether there were any abnormalities in the participants’ physical condition and asked them verbally about the presence or absence and degree of these symptoms. None of the participants exhibited any symptoms of severe VR sickness.

### Questionnaire

#### Validity of the VR environment

To examine the validity of the VR-SC environment, the following were measured at the Post time point. Sense of presence in the VR space was measured using the scale used by Slater et al.^26^. The questionnaire consisted of seven items rated on a seven-point scale from −3 (least agreement) to 3 (most agreement).

For the body ownership illusion and sense of agency, we used the scale used by Slater et al.^26^ with the addition of 3D avatar similarity items used by Osimo et al.^25^ and modified the questionnaire in a manner compatible with the procedures in our study. The questionnaire consisted of nine items rated on a seven-point scale from − 3 (least agreement) to 3 (most agreement).

In addition, the degree of perspective-taking of the counseling partner was rated on an 11-point scale from 0 (not at all possible) to 10 (very possible).

### Outcome measures

#### Discomfort level of the problem

Participants were asked to rate the degree of discomfort with the problems addressed in this study on an 11-point scale ranging from 0 (peace, serenity) to 10 (insupportable). The measurement points were the BL, Pre, Post, and FU time points.

#### Depressive and anxiety symptoms

The Japanese version of the Patient Health Questionnaire-9 (PHQ-9^28,29^) was used to measure the degree of depressive symptoms. The questionnaire consisted of nine items rated on a four-point scale from 0 (not at all) to 3 (nearly every day). This scale calculates the total score of each question item, and the higher the score, the more severe the level of depressive symptoms. The reliability and validity of this scale have been shown in many previous studies, and in the data of participants in our study, the alpha coefficient was confirmed to be adequate (Cronbach’s *α* = 0.795).

The Japanese version of the Generalized Anxiety Disorder-7 (GAD-7^30,31^) was used to measure the severity of anxiety symptoms. The questionnaire consisted of seven items rated on a four-point scale from 0 (not at all) to 3 (nearly every day). This scale calculates the total score of each question item, and the higher the score, the more severe the level of anxiety symptoms. The reliability and validity of this scale have been shown in many previous studies, and in the data of participants in our study, the alpha coefficient was confirmed to be adequate (Cronbach’s *α* = 0.768).

These questionnaires were administered at BL, Pre, and FU time points.

### Outcome measures related to the problem

As outcome measures for the problems, we used some of the measures used by Slater et al.^26^. The questions were modified to fit the measurement time point and intervention method so that the intent of the questions could be conveyed to the participants. Table 1 lists the questions used in our study along with the measurement time points.

### Change in problems and impressions during the intervention and rest period

We asked the participants whether there were changes in their problems due to the intervention or rest period at two time points: Post and FU. If there were changes in their problems, participants were asked to respond in the form consisting of an open-ended question about the details and reason for the change. In addition, at the same two time points, participants were asked about their impressions of the intervention or rest period in an open-ended form.

### Data analyses

The statistical package for the social sciences (SPSS) version 24 (IBM, Tokyo, Japan) was used for the statistical analyses, and the level of significance was set at *p* < 0.05. Regarding the validity of the VR environment, to examine the difference in the degree of perspective-taking of the counseling partner between the IP and Freud groups, a *t*-test was conducted. To examine the effects of the intervention, two-way analysis of variance (ANOVA) tests were performed for each outcome measure, with the groups as between-subject factors and measurement time points as within-subject factors.

### Supplementary Information

Supplementary Information.

## Data Availability

The datasets used and/or analyzed during the current study are available from the corresponding author on reasonable request.
