# Supplementary material for: Effect of virtual reality self-counseling with the intimate other avatar
Source: Sci Rep. 2024 Jul 4;14:15417. doi: 10.1038/s41598-024-65661-6 (PMC11224315; doi:10.1038/s41598-024-65661-6)

**Effect of Virtual Reality Self-Counseling with the Intimate Other Avatar**

**Supplementary Materials**

**Contents**

[Supplementary Table S1. The content of the problems addressed in the study 2](#_Toc149572228)

[Supplementary Table S2. Mean and standard deviation for each question item in the sense of presence scale 3](#_Toc149572229)

[Supplementary Table S3. Mean and standard deviation for each question item in the body ownership illusion and sense of agency scale 3](#_Toc149572230)

[Supplementary Table S4. Change of the problems in each group at Post time point 4](#_Toc149572231)

[Supplementary Table S5. Change of the problems in each group at FU time point 4](#_Toc149572232)

[Supplementary Figures S1-6. Graphs of outcome measures in Table 3 5](#_Toc149572233)

[Supplementary Figure S7. Guidance session 8](#_Toc149572234)

[Supplementary Figure S8. VR-SC with Freud 8](#_Toc149572235)

[Supplementary Results. Outcome measures related to the problems addressed in the study 9](#_Toc149572236)

[Supplementary Methods. Equipment used in the study and treatments performed 9](#_Toc149572237)

[Supplementary Movie. Overview of VR-SC 10](#_Toc149572238)

**Supplementary Table S1. The content of the problems addressed in the study**

| Group | Content | *n* |
| --- | --- | --- |
| VR-SC with intimate persons | Concerns about course or future | 5 |
|  | Concerns about my character | 4 |
|  | The habit of procrastination | 2 |
|  | Concerns about study | 1 |
|  | Concerns about graduation thesis or master's thesis | 1 |
|  | Concerns about club activities | 1 |
|  | Concerns about family relationship | 1 |
|  | Concerns about marriage | 1 |
|  | Concerns about money | 1 |
|  | Concerns about communication | 1 |
|  | The habit I want to fix | 1 |
|  | I sometimes find it hard to stay alive | 1 |
| VR-SC with Freud | Concerns about course or future | 6 |
|  | Concerns about human relationship | 4 |
|  | Concerns about my character | 2 |
|  | Concerns about club activities | 2 |
|  | Concerns about study | 1 |
|  | Concerns about romance | 1 |
|  | Concerns about communication | 1 |
|  | Anxiety about the opposite sex | 1 |
|  | Complaints about the coronavirus crisis | 1 |
|  | Ascertainment behavior | 1 |
| Control | Concerns about course or future | 6 |
|  | Concerns about graduation thesis or master's thesis | 3 |
|  | Concerns about study | 2 |
|  | Concerns about my character | 2 |
|  | Concerns about university life | 2 |
|  | Concerns about human relationship | 1 |
|  | Concerns about money | 1 |
|  | The habit of procrastination | 1 |
|  | Concerns about time management | 1 |
|  | Emotional instability | 1 |

**Supplementary Table S2. Mean and standard deviation for each question item in the sense of presence scale**

| **Variable** | **Questionnaire item** | **VR-SC with intimate persons group（*n* = 20）** | **VR-SC with Freud group （*n* = 20）** |
| --- | --- | --- | --- |
| *There* | I had the sensation being there seated in the virtual counseling room (+3 corresponds to the normal sensation of being in a place). | 1.90（0.64） | 1.90（0.79） |
| *Visited* | When you think about your experience, do you remember the virtual counseling as some images that you have seen or as a place where you have been? | 1.95（1.10） | 1.30（1.49） |
| *Together* | I had the feeling of sharing the virtual counseling with the other person, as if we were really in the same place. | 1.30（1.17） | 1.20（1.28） |
| *Real Conversation* | I had the feeling that the conversation between the other person and me was really happening. | 0.85（1.18） | 0.80（1.61） |
| *Emotion* | My emotional response was the same as in a real situation. | 1.65（1.14） | 1.60（1.31） |
| *Behavior* | My behavior was the same as in a real situation. | 1.70（1.03） | 1.45（1.47） |
| *Thoughts* | My thoughts in relation to the conversation were the same as in a real situation. | 2.00（1.30） | 1.95（1.39） |

**Supplementary Table S3.** **Mean and standard deviation for each question item in the body ownership illusion and sense of agency scale**

| **Variable** | **Questionnaire item** | **VR-SC with intimate persons group（*n* = 20）** | **VR-SC with Freud group （*n* = 20）** |
| --- | --- | --- | --- |
| *Self Recognition* | Did you recognize yourself in the virtual body that was sitting in front of your counseling partner? | 1.55（1.23） | 1.40（1.14） |
| *OwnDown* | When I was sitting in front of my counseling partner: I felt that the virtual body I saw looking down was my own body. | 1.70（1.03） | 1.80（1.06） |
| *OwnMirror* | When I was sitting in front of my counseling partner: I felt that the virtual body that I saw when I looked towards the mirror was my own body. | 1.25（1.33） | 1.20（1.61） |
| *OwnAgency* | When I was sitting in front of my counseling partner: I felt that the movements of the virtual body were caused by my own movements. | 1.55（1.15） | 1.80（1.24） |
| *Counselor Down* | When I was in my counseling partner's body: Although the virtual body did not look like me physically, I felt that the virtual body I saw looking down was my own body. | 1.00（1.38） | 1.10（1.41） |
| *Counselor Mirror* | When I was in my counseling partner's body: I felt that the virtual body that I saw when I looked towards the mirror was my own body. | 1.25（1.25） | 0.80（1.44） |
| *Counselor Agency* | When I was in my counseling partner's body: I felt that the movements of the virtual body were caused by my own movements. | 1.35（1.46） | 1.55（1.19） |
| *LikeMe* | My body in the virtual world looked like my real body. | 2.25（1.07） | 2.15（1.14） |
| *Like Counselor* | The body of my counseling partner in the virtual world looked like their real body. | 2.35（1.23） | 1.00（1.56） |

**Supplementary Table S4. Change of the problems in each group at Post time point**

| **Question** | **VR-SC with intimate persons group（*n* = 20）** | **VR-SC with Freud group （*n* = 20）** | **Control group （*n* = 20）** |
| --- | --- | --- | --- |
| Before and after this counseling (rest period), did anything change regarding your problem? | Yes: 20 No: 0 | Yes: 18 No: 2 | Yes: 7 No: 13 |
| Are you doing, feeling or thinking about the problem differently from the way you did before? | Yes: 20 No: 0 | Yes: 18 No: 2 | Yes: 5 No: 15 |

| **Question** | **VR-SC with intimate persons group（*n* = 20）** | **VR-SC with Freud group （*n* = 20）** | **Control group （*n* = 20）** |
| --- | --- | --- | --- |
| During your participation in this study (through the three visits), did anything change regarding your problem? | Yes: 20 No: 0 | Yes: 19 No: 1 | Yes: 11 No: 9 |
| Are you doing, feeling or thinking about the problem differently from the way you did before (before participating in the study)? | Yes: 20 No: 0 | Yes: 19 No: 1 | Yes: 8 No: 12 |

**Supplementary Table S5. Change of the problems in each group at FU time point**

To the first question, 12 participants in the control group answered "Yes,” but one of them reported that the change was not due to their participation in the study. In addition, 10 participants in the control group answered "Yes" to the second question, but two of them reported that the change was not due to their participation in the study.

**Supplementary Figures S1-6. Graphs of outcome measures in Table 3**

**Supplementary Figure S1. Line graph of means and standard errors of PHQ-9 (Patient Health Questionnaire-9)**

**
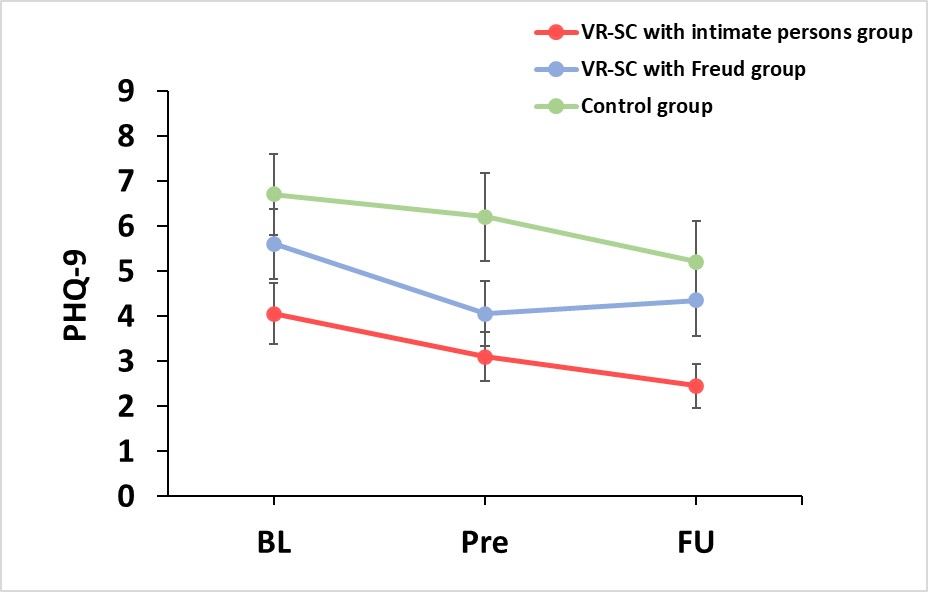
**

**Supplementary Figure S2. Bar graph of means and standard errors of The *Understand* variable**


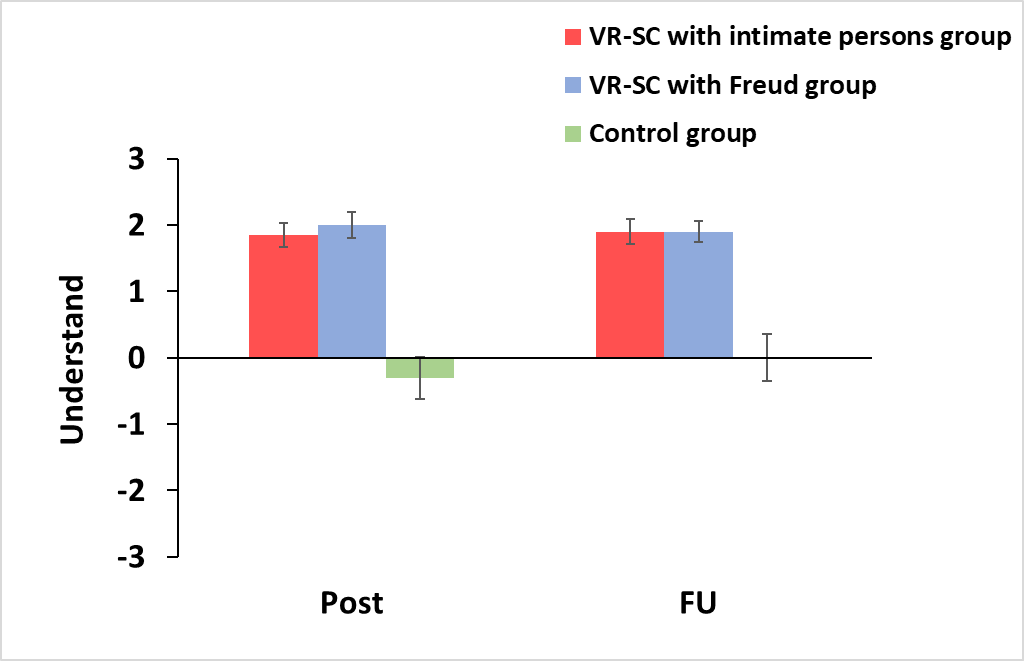


**Supplementary Figure S3. Bar graph of means and standard errors of The *NewIdeas* variable**


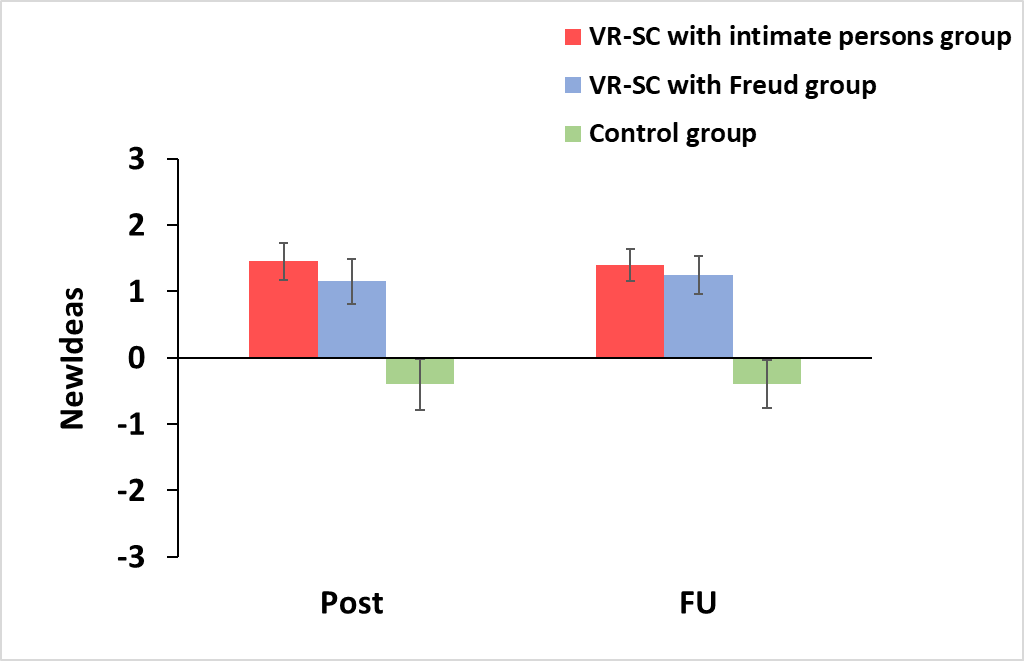


**Supplementary Figure S4. Bar graph of means and standard errors of The *BetterControl* variable**


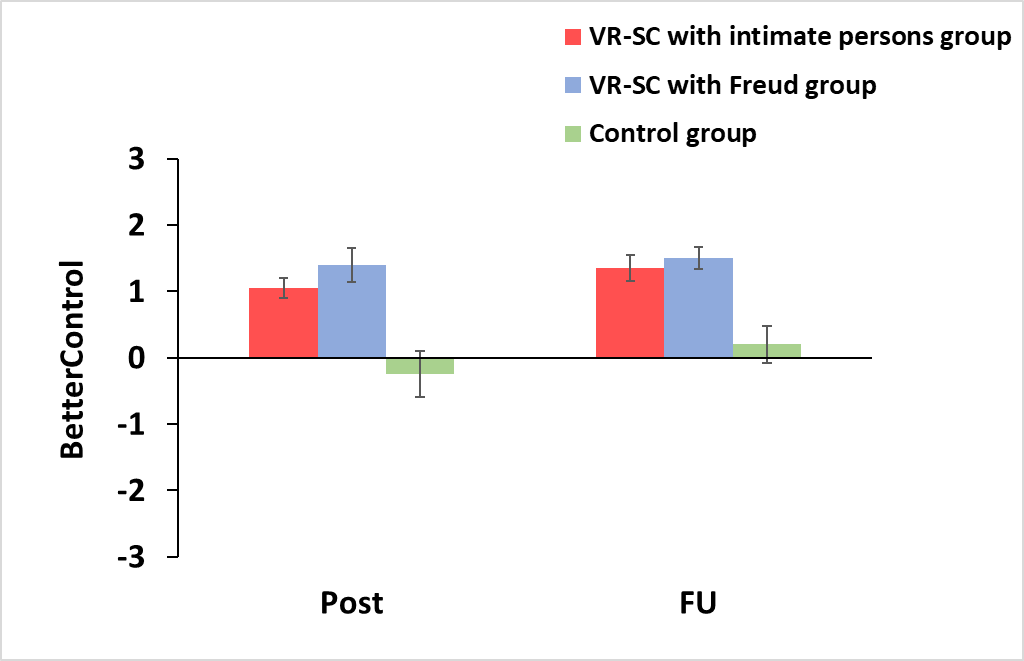


**Supplementary Figure S5. Bar graph of means and standard errors of The *Helped* variable**


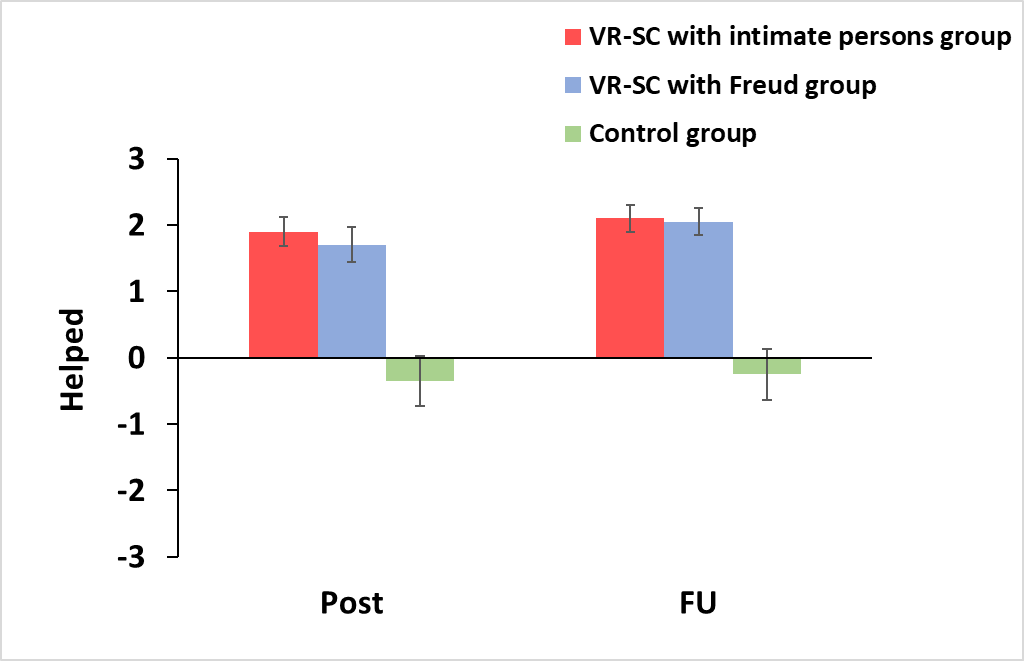


**Supplementary Figure S6. Bar graph of means and standard errors of the degree of perspective-taking of the counseling partner**


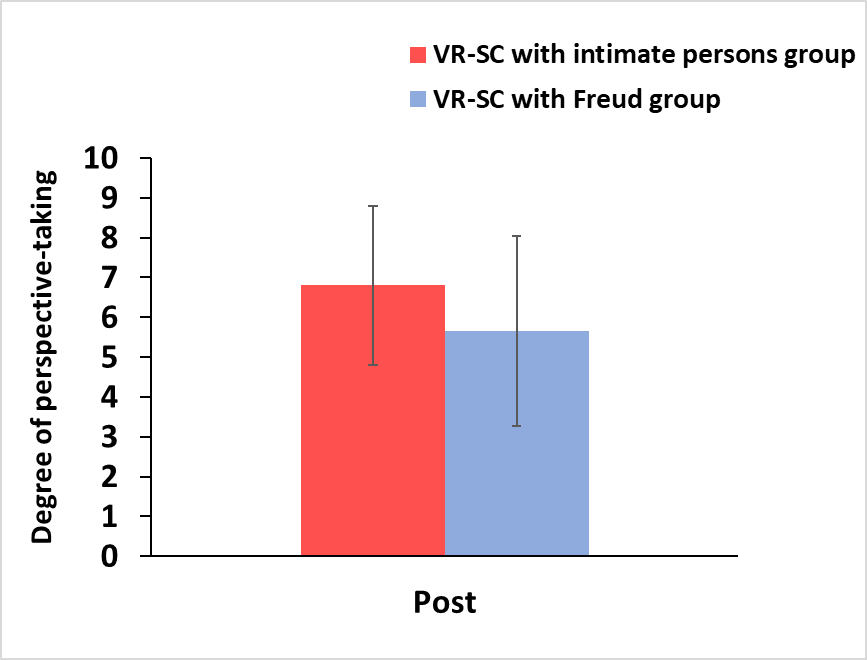


**
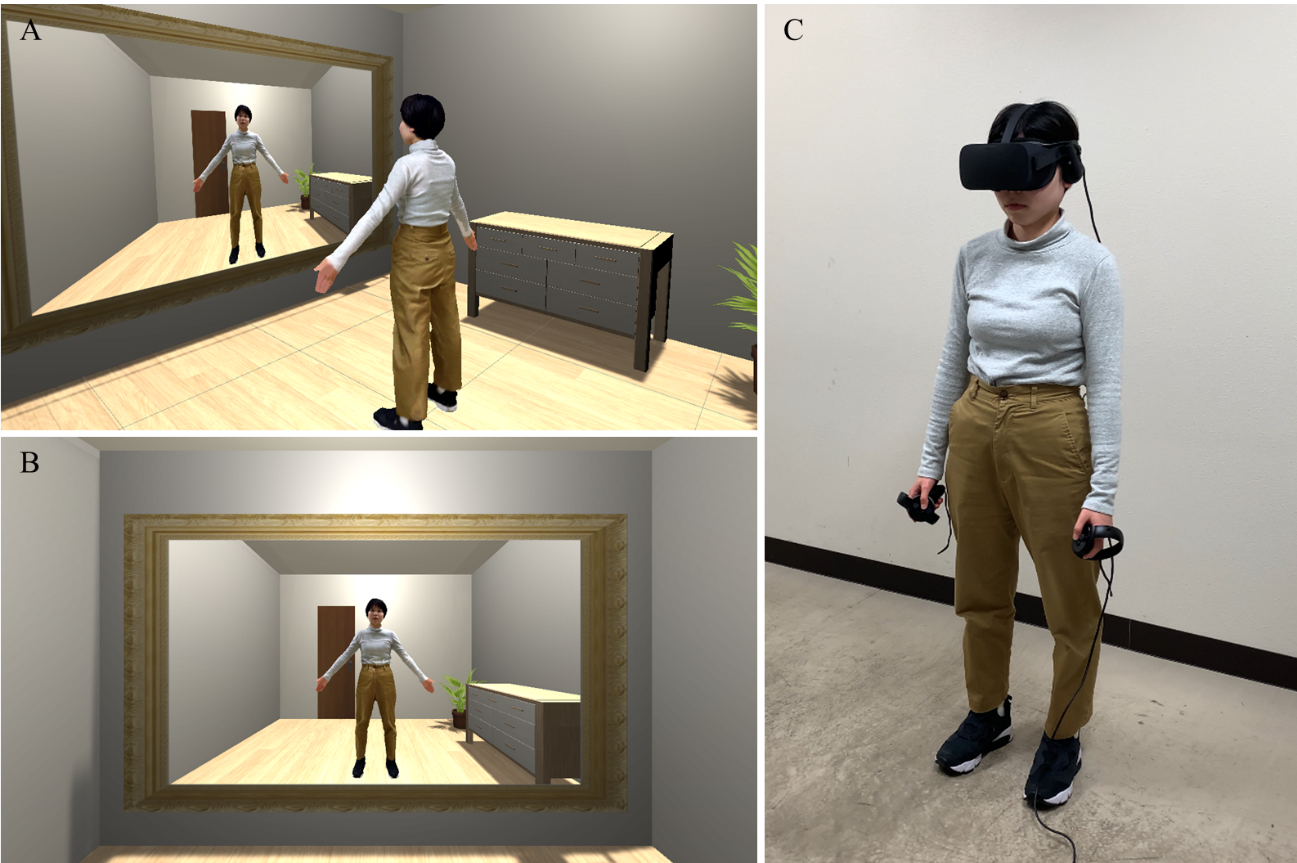
Supplementary Figure S7.** **Guidance session**

(A) Overhead view of the VR space for the guidance session. (B) VR space from the viewpoint of a participant. (C) The participant during the guidance session.

**
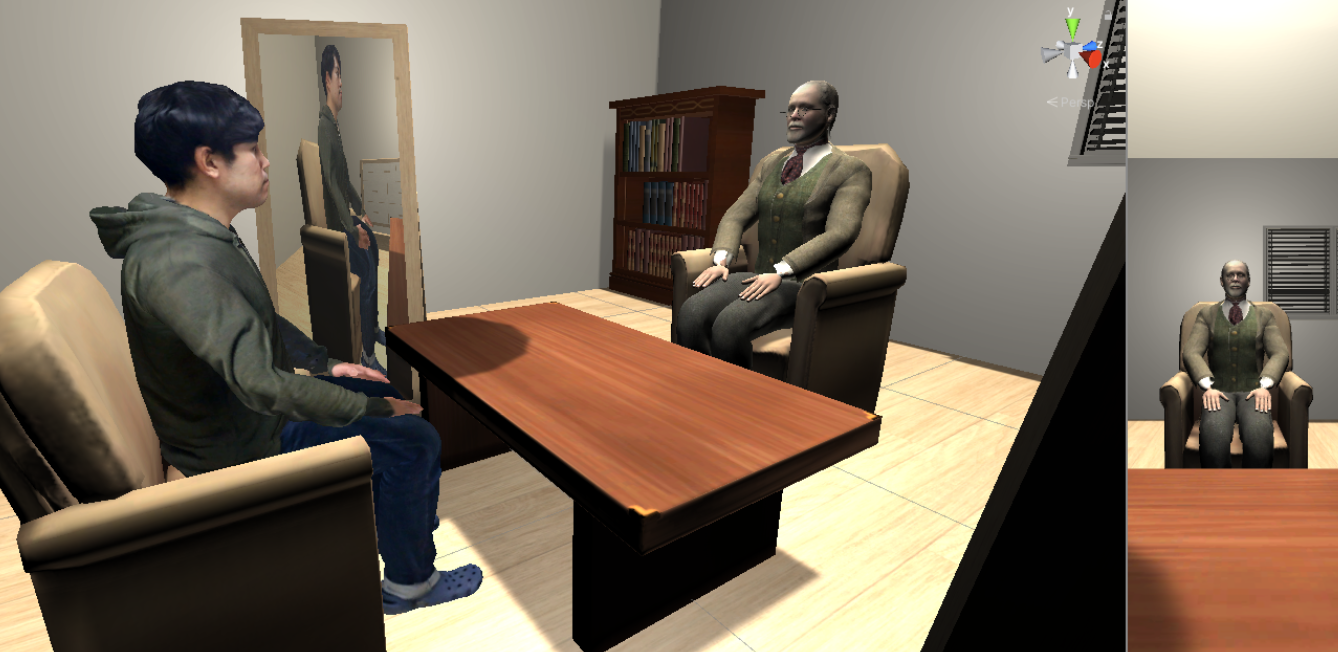
Supplementary Figure S8. VR-SC with Freud**

**Supplementary Results. Outcome measures related to the problems addressed in the study**

The results of the outcome measures for which no significant interactions were found are as follows. The *Understand, NewIdeas,* and *Helped* variables did not have a main effect of time (*F* (1, 57) ≤ 2.70, *p* ≥ .106, *ηp^2^* ≤ .045), and the interaction between group and time was not significant (*F* (2, 57) ≤ .621, *p* ≥ .541, *ηp^2^* ≤ .021), but the main effect of group was significant (*F* (2, 57) ≥ 12.39, *p* < .001, *ηp^2^* ≥ .303). Multiple comparisons were made for each of the main effects of the groups, and scores of the VR-SC with intimate persons and Freud groups were significantly higher than those of the control group (*ps* < .001). The *BetterControl* variable also had no interaction (*F* (2, 57) = .60, *p* = .554, *ηp^2^* = .020), but the main effects of group (*F* (2, 57) = 13.56, *p* < .001, *ηp^2^* = .322) and time (*F* (1, 57) = 4.66, *p* = .035, *ηp^2^* = .076) were significant. Multiple comparisons of the main effect of the groups showed that the scores of the VR-SC with intimate persons and Freud groups were significantly higher than those of the control group (*ps* < .001).

**Supplementary Methods. Equipment used in the study and treatments performed**

For body scanning to create 3D avatars, we attached a 3D scanner, Structure Sensor Mark II, to an iPad 6th generation, and used itSeez3D, an app for 3D scanning, to capture full-body images of the participants and their intimate others. During the scan, we asked participants to stand with their arms in the A pose. After scanning, the 3D data were sculpted and painted using Blender, an integrated 3DCG software, to improve avatar accuracy. The avatar was then rigged using Mixamo, a web service for creating animations of 3D models, and Blender was again used to make minor modifications to the avatar's mesh and create animations (standing, sitting, sitting with breathing motion, and sitting with nodding motion). In VR-SC, the avatar in front of the participants was animated as if it were breathing, so that the experience in the VR space was felt similar to the real world. Furthermore, to enhance the sense that the counseling partner was listening to them, we set up a system so that nodding motions were periodically played to the avatar of the counseling partner when the avatar was in front of them and they were talking about their own problems from their own avatar. For Freud's 3D avatar, we obtained permission from the creator and used the avatar posted on Sketchfab, a 3D model-sharing platform.

To create the VR space, Unity 2019.3.12f1 was used. Using this, we created a space for a guidance session (Supplementary Figure S7) to generate the body ownership illusion, and a space for a VR-SC session to conduct counseling (Figure 4, Supplementary Figure S8). The 3D avatars created using the above procedure were imported into these spaces.

The head-mounted display (HMD) was an Oculus Rift CV1. The resolution was 2160 × 1200 (1080 × 1200 for one eye), refresh rate was 90 Hz, viewing angle was 110°, and weight was approximately 470 g. The Oculus Touch was used as the controller, and the Oculus Sensor was used to track the HMD and two-handed controller. The tracking information was mapped to the avatar's movements using the inverse kinematics (IK) technique.

A laptop computer, OMEN 17 (CPU: Intel Core i9-9880H, RAM:32GB, graphics board: NVIDIA GeForce RTX 2080, OS: Windows 10 Pro 64bit, resolution:1920×1080), was used to run the computer programs.

# **Supplementary Movie. Overview of VR-SC**

The link to a movie explaining the VR-SC procedure is below.

<https://youtu.be/Yt6RF_yYo1Y>


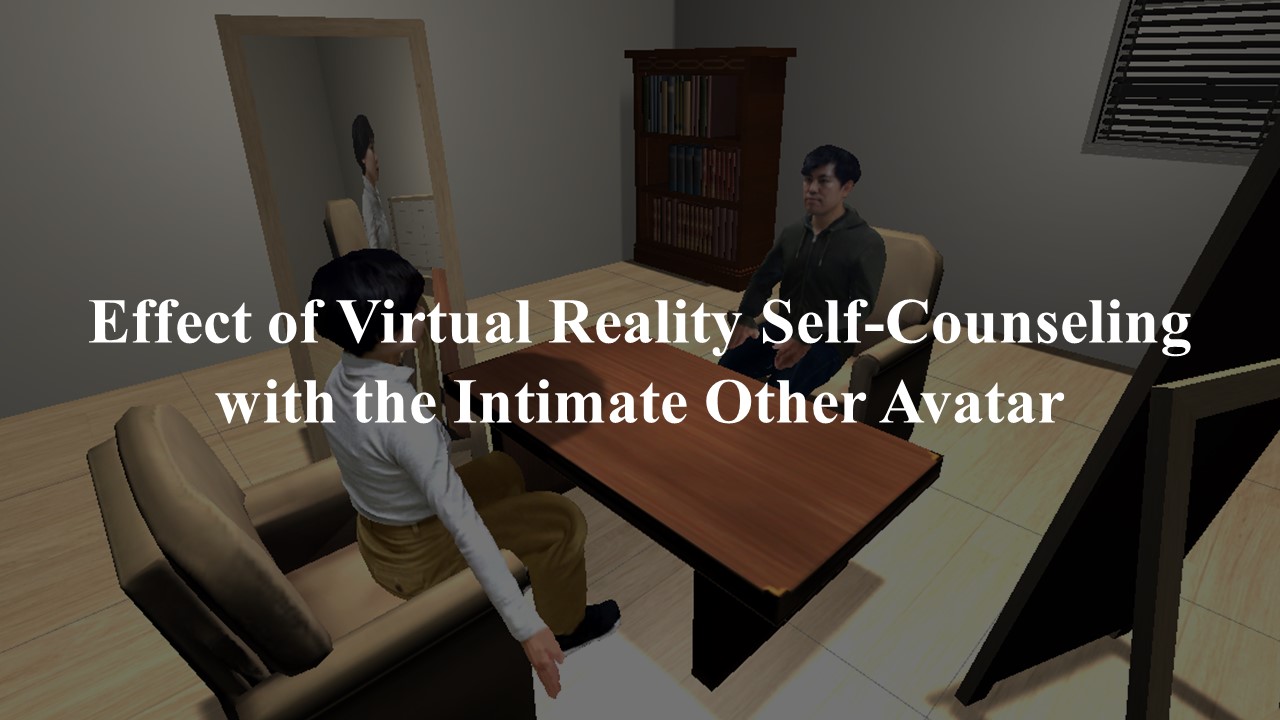

Supplement: Supplementary file 1 — Supplementary Information. [file 41598_2024_65661_MOESM1_ESM.docx]
